# Cytogenomic Investigation of Syndromic Brazilian Patients with Differences of Sexual Development

**DOI:** 10.3390/diagnostics13132235

**Published:** 2023-06-30

**Authors:** José Antonio Diniz Faria, Daniela R. Moraes, Leslie Domenici Kulikowski, Rafael Loch Batista, Nathalia Lisboa Gomes, Mirian Yumie Nishi, Evelin Zanardo, Carolina Kymie Vasques Nonaka, Bruno Solano de Freitas Souza, Berenice Bilharinho Mendonca, Sorahia Domenice

**Affiliations:** 1 Faculdade de Medicina, Universidade Federal da Bahia, Salvador 40110-909, Brazil; joseantonio.endocrinoped@gmail.com; 2 Unidade de Endocrinologia do Desenvolvimento, Laboratório de Hormônios e Genética Molecular LIM/42, Hospital das Clínicas da Faculdade de Medicina da Universidade de São Paulo, São Paulo 05403-010, Brazil; danielar.moraes@gmail.com (D.R.M.); rafael.loch@hc.fm.usp.br (R.L.B.); nathalialisboa.endocrino@gmail.com (N.L.G.); minishi@usp.br (M.Y.N.); beremen@usp.br (B.B.M.); 3 Laboratório de Citogenômica e Patologia Molecular LIM/03, Hospital das Clínicas da Faculdade de Medicina da Universidade de São Paulo, São Paulo 05403-010, Brazil; lesliekulik@gmail.com (L.D.K.); evelinzanardo@yahoo.com.br (E.Z.); 4 Centro de Biotecnologia e Terapia Celular, Hospital São Rafael, Salvador 41253-190, Brazil; carolina.nonaka@hsr.com.br (C.K.V.N.); bruno.solano@fiocruz.br (B.S.d.F.S.); 5 Instituto D’Or de Pesquisa e Ensino (IDOR), Salvador 41253-190, Brazil; 6 Instituto Gonçalo Moniz, Fundação Oswaldo Cruz (FIOCRUZ), Salvador 40296-710, Brazil

**Keywords:** disorders of sexual development, copy number variation, SNP array

## Abstract

Background: Cytogenomic methods have gained space in the clinical investigation of patients with disorders/differences in sexual development (DSD). Here we evaluated the role of the SNP array in achieving a molecular diagnosis in Brazilian patients with syndromic DSD of unknown etiology. Methods: Twenty-two patients with DSD and syndromic features were included in the study and underwent SNP-array analysis. Results: In two patients, the diagnosis of 46,XX *SRY +* DSD was established. Additionally, two deletions were revealed (3q29 and Xp22.33), justifying the syndromic phenotype in these patients. Two pathogenic CNVs, a 10q25.3-q26.2 and a 13q33.1 deletion encompassing the *FGFR2* and the *EFNB2* gene, were associated with genital atypia and syndromic characteristics in two patients with 46,XY DSD. In a third 46,XY DSD patient, we identified a duplication in the 14q11.2-q12 region of 6.5 Mb associated with a deletion in the 21p11.2-q21.3 region of 12.7 Mb. In a 46,XY DSD patient with delayed neuropsychomotor development and congenital cataracts, a 12 Kb deletion on chromosome 10 was found, partially clarifying the syndromic phenotype, but not the genital atypia. Conclusions: The SNP array is a useful tool for DSD patients, identifying the molecular etiology in 40% (2/5) of patients with 46,XX DSD and 17.6% (3/17) of patients with 46,XY DSD.

## 1. Introduction

Disorders/differences of sexual development (DSDs) affect 1:1000–4500 live births, encompassing a wide spectrum of abnormalities that are secondary to atypical chromosomal, gonadal, or genital sex development [1,2,3,4]. Although early diagnosis of DSD patients is essential for a better prognostic assessment, therapeutic guidance, reproductive planning, and genetic counseling, in a significant percentage of affected individuals the etiology cannot be established by classical methods of investigation [5,6]. 

Molecular genetics and cytogenomics have contributed to establishing a molecular diagnosis in 46,XY DSD and 46,XX DSD patients with unknown etiology [7,8,9]. A single nucleotide polymorphism array (SNP array) may identify submicroscopic changes in the whole genome, recognizing the deleted or the duplicated genomic segments larger than 1 Kb, and confirming the variation in the number of gene copies (CNVs) [6]. 

Microarray techniques have been proposed as the gold standard method to investigate syndromic DSD patients. However, the utility of such methods in clinical practice is not well established since most of the published data rely on a small number of patients and almost exclusively Caucasian/Asian populations, with a low representative sample of Latin American and Afro-descendant populations. A cohort of 22 Brazilian patients with syndromic DSD of unknown etiology was studied to evaluate the role of the SNP-array analysis to achieve a final molecular diagnosis.

## 2. Materials and Methods

### 2.1. Ethical Approval

The study was conducted according to the ethical principles following the Declaration of Helsinki. The project was approved by the Ethics Committee for Analysis of Research Projects of the Hospital das Clínicas da Faculdade de Medicina da Universidade de São Paulo (HC-FMUSP) under the approval number CAAE: 60700616.9.0000.0068. Written consent was obtained from all patients or legal guardians before the research procedures were initiated.

### 2.2. Study Design

A case series that included patients referred to the outpatient clinic of the Development Endocrinology Unit at the University of São Paulo between 2016 and 2020 and is summarized in Table 1. The inclusion criteria were as follows: DSD diagnosis with atypical genitalia at birth in association with other malformations not associated with the genitourinary system; the etiological diagnosis had not been established after hormonal, classical cytogenetic analysis; and imaging studies.

### 2.3. DNA Extraction, Polymerase Chain Reaction (PCR) Technique, and Multiplex Ligation-Dependent Probe Amplification (MLPA) Technique

Genomic DNA was extracted from peripheral blood leukocytes using the proteinase K–SDS salting-out method [10]. The PCR technique was used to confirm the presence of Y chromosome fragments in the genomic DNA of 46,XX DSD patients [11]. We searched for the presence or absence of the SRY (p11.31), TSPY (p11.2), AMELY (p11.2), DYZ3 (centromere), DYS280 (q11.22), and DYS1 (q11.22) genes as previously described [11]. 

DNA samples were investigated by MLPA with the SALSA MLPA probemixes P036-E1 Human Telomere-3 (MRC-Holland) and SALSA MLPA P095-A3 Aneuploidy probemix (MRC-Holland) kits, which were used according to the manufacturer’s recommendations (MRC-Holland^®^, Amsterdam, The Netherlands). Kit P036-E1 was used to detect CNVs within the 3q29 region (especially the BDH1) and kit P095-A3 was used to detect regions in Yp11.31 (SRY and ZFY) and Yq11.221 (UTY) regions. MLPA results were considered altered when the relative peak was lower than 0.75 (deletion) or higher than 1.25 (duplication) when compared to the control results. 

### 2.4. Array Analysis

A genomic array analysis was performed for 15 patients using the Infinium CytoSNP-850K BeadChip^®^ (Illumina^®^, Washington, DC, USA) and 7 patients using the Affymetrics CytoScan HD 750 K (Affymetrix, Santa Clara, CA, USA) according to the manufacturer’s instructions. These probes targeting all regions of known cytogenetic importance cover the entire genome with probe medium spacing of 1.8 Kb and 1.1 Kb for the Illumina and the Affymetrics array platforms, respectively. Data analysis was performed using BlueFuse Multi 4.3^®^ (BlueGnome^®^, Illumina Inc. Washington, DC, USA) and Chromosome Analysis Suite (ChAS) (Affymetrix Inc., Santa Clara, CA, USA), respectively, for the Illumina and Affymetrics arrays. Only deletions or duplications that compromised the consecutive hybridization of at least 10 probes were reported and analyzed in both methods. An additional filter was necessary for the Affymetrics array in the following manner: 2.5 Kb of minimal size for deletions and 5 Kb for duplications.

The genomic imbalances were annotated based on the Genome Reference Consortium Human Build 37 (GRCh37)/hg19 URL (accessed on October 2021) (http://www.ncbi.nlm.nih.gov/projects/genome/assembly/grc/). 

### 2.5. CNV Classification 

The identified CNVs were analyzed according to the American College of Medical Genetics and Genomics (ACMG) criteria [12] and were compared with the following databases of genomic variation data: Database of Genomic Variants URL (accessed on October 2021) (DGV, http://projects.tcag.ca/variation); Database of Chromosomal Imbalance and Phenotype in Humans Using Ensemble Resources URL (accessed on October 2021) (DECIPHER; http://decipher.sanger.ac.uk/); the International Standard Cytogenomic Array URL (accessed on October 2021) (ISCA; https: //www.iscaconsortium.org/); Clinically Relevant Variants URL (accessed on October 2021) (CLINVAR, https://www.ncbi.nlm.nih.gov/clinvar/); University of California, Santa Cruz Genome Browser URL (accessed on October 2021) (UCSC, http://genome.ucsc.edu/); Online Mendelian Inheritance for Man URL (accessed on October 2021) (OMIM, https://www.omim.org); and PubMed URL (accessed on October 2021) (https://www.ncbi.nlm.nih.gov/pubmed/). Chromosomal region, CNV content, and CNV classification can be found in Table 2. A semi-quantitative web-based CNV classification calculator was also used to verify the pathogenicity URL (accessed on October 2021) (http://cnvcalc.clinicalgenome.org/cnvcalc/).

## 3. Results

### 3.1. Clinical Characteristics of the Syndromic DSD Patients

Twenty-two patients with DSD in association with multiple malformations were included in the study. A 46,XY karyotype was present in 16 patients, 46,XX in 5 patients, and 47,XYY in 1 patient (Table 1). Karyotype analysis revealed chromosomal aberrations in three patients: patient #7 (46,XY del 10q); patient #13 (46,XY del 1q); and patient #14 with a balanced translocation 46,XY, t (3; 9). 

Eight patients were born premature or small for gestational age (SGA). Limb malformations were observed in all patients. Seven patients presented with congenital heart disease and two of them had been previously submitted for cardiac surgical correction. Five patients had anorectal malformations; renal and central nervous system malformations were found in six patients. All patients presented neuropsychomotor developmental delay (NPMD) and two patients were obese. External genitalia masculinization scores (EMS) and Prader genital scale values are shown in Table 1 [3,13]. 

### 3.2. CNVs

A total of 229 CNVs were identified in the 22 patients studied, with an average of 10.3 CNVs per patient. The size of the CNVs ranged from 2.8 Kb to 44.5 Mb. Among the CNVs, 126 were deletions, 66 duplications, and 37 regions with loss of heterozygosity (LOH). Two patients (#12 and #16) carried 31 of the 37 LOH, both with parental history of consanguinity. 

After a careful analysis, nine CNVs in six patients (6/22; 27.2%) were considered pathogenic or likely pathogenic. Sixty-five CNVs were considered variants of uncertain significance (VUS), and 155 were classified as benign or likely benign (Table 2). 

### 3.3. Relationship between Clinical Diagnosis and SNP-Array Results

**Figure 1:**
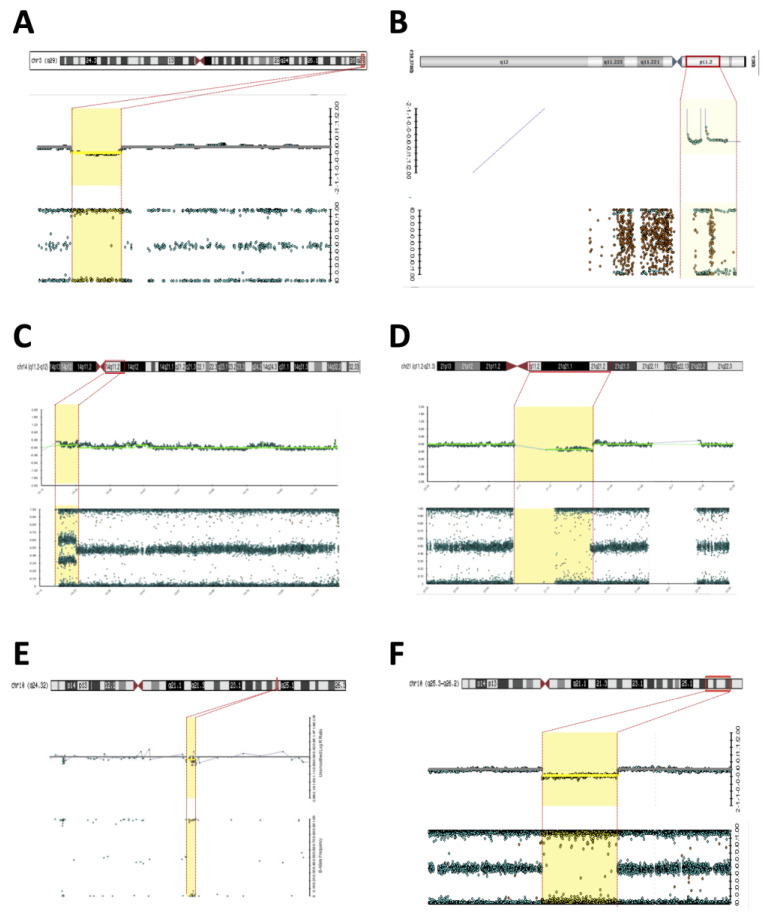
Microarray (Illumina) rearrangements detected in syndromic DSD patients. Detected chromosomal regions (red bar) in patients are shown in the top section followed by the signal intensity and B-allele frequency for each CNV (yellow dashed). (**A**) 3q29 deletion in patient 2; (**B**) Yp11.31 gain in patient 2; (**C**) 14q11.2-q12 duplication in patient 15; (**D**) 21p11.2-q21.3 deletion in patient 15; (**E**) 10q24.32 deletion in patient 6; (**F**) 10q25.3-q26.2 deletion in patient 7.

**Figure 2:**
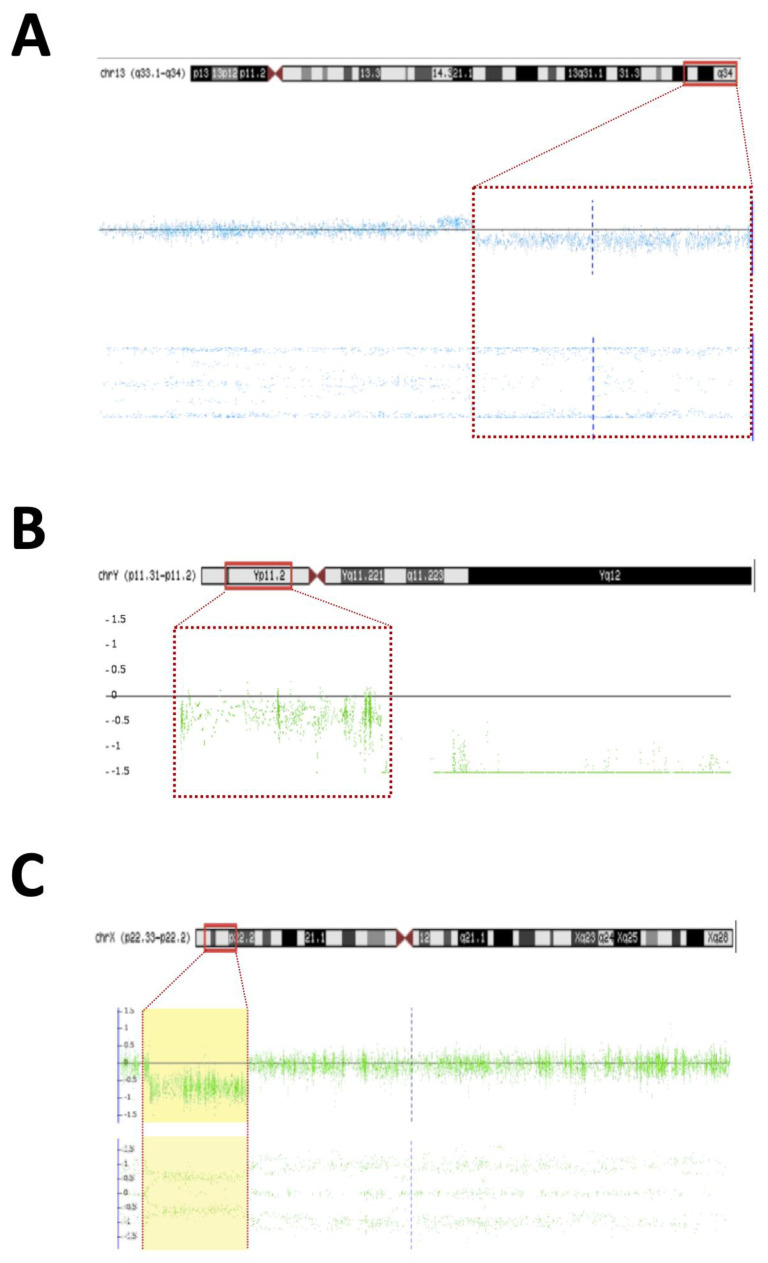
Microarray rearrangements (Affymetrics) detected in syndromic DSD patients. Detected chromosomal regions (red bar) in patients are shown in the top section followed by the signal intensity and B-allele frequency for each CNV (yellow dashed). (**A**) 13q33.1q34 deletion in patient 22; (**B**) Yp11.31 gain in patient 5; (**C**) Xp22.33 deletion in patient 5.

- **Patient #2**—An 8-year-old boy without genital ambiguity was referred to the genetics service due to school difficulties, language delay, epilepsy, and the presence of dysmorphic facial and body features (Table 1). A brain MRI revealed bilateral and nonspecific periventricular leukomalacia. The EKG and abdominal and pelvic ultrasounds were normal. Cytogenetic investigation revealed a 46,XX karyotype. The SNP array allowed clarification of the karyotype and revealed a 7 Mb Yp11.31 chromosome fragment containing the *SRY* and a 1.7 Mb deletion in the 3q29 region (Figure 1A,B). The presence of Y-chromosome material in patient #2 was confirmed using the MLPA technique, which identified the genes located in Yp11.31 (*SRY* and *ZFY*) and the absence of the *UTY* located in Yq11.22. The patient displayed clinical features suggestive of 3q29 deletion syndrome (OMIM #609425) and the MLPA testing confirmed the deletion of the exon 4 in the BDH1 located in the 3q29. The analysis of parental samples showed no abnormalities in chromosome 3q. The diagnosis of syndromic 46,XX testicular DSD *SRY (+)* associated with 3q29 deletion syndrome was proposed.
- **Patient #5**—A 30-year-old woman was referred to the Endocrinology Unit due to a partial lack of pubertal development. In her childhood, the diagnosis of Fraser syndrome (OMIM #219000) was made based on the presence of microphthalmia with bilateral amaurosis associated with virilization of the external genitalia (Table 1). In adulthood, she was obese (BMI—31.5 kg/m^2^), and had NPMD with epilepsy along with microphthalmia and amaurosis. Clitoromegaly (4.0 × 2.0 cm), asymmetric partial labial fusion, single perineal orifice, and palpable gonad in the right inguinal canal and non-palpable left gonad were observed. A pelvic MRI ruled out Mullerian derivatives and gonads were located in the inguinal canal bilaterally. A brain MRI revealed agenesis of the corpus callosum and pellucid septum, volumetric reduction of the temporal lobes and hippocampus, dilation of the ventricular system, and hypotrophy of the eyeballs. At the age of 30, she was hypogonadal (low testosterone and estrogen levels), with inappropriately normal gonadotropins. She was diagnosed with 46,XX DSD due to abnormal gonadal development, and hypogonadism associated with ocular and neurological malformations and convulsive phenotype. A 46,XX karyotype without abnormalities and two pathogenic CNVs were identified in SNP-array analysis. A 7.1 Mb Yp11.31 chromosome fragment containing the *SRY* caused her atypical genitalia and the 9.1 MB deletion located at Xp22.33 was associated with the patient’s syndromic features (Figure 2B,C). A new diagnostic hypothesis was proposed based on the clinical features of the patient associated with the Xp22.33 deletion, which was previously associated with Aicardi syndrome.
- **Patient #6**—A 2-year-old boy with atypical genitalia (normal penile length, proximal hypospadias, bifid scrotum, and bilateral cryptorchidism) without Mullerian duct remnants was seen at the Endocrinology Unit. The patient underwent surgical correction of the genitalia (orthophalloplasty with neourethroplasty and subsequent correction of urethral fistula) as well as bilateral orchiopexy at 5 years of age. The patient also had congenital cataracts and epilepsy associated with mild speech delay and lower than expected school performance for the age group. During the etiological investigation, a 46,XY karyotype without abnormalities was obtained and testosterone after hCG stimulus test was normal and without androgen precursors’ accumulation. Androgen receptor (*AR*) gene sequencing was normal. SNP-array analysis identified a 12 Kb deletion at 10q24.32 encompassing the *PITX3* gene (Figure 1E). *PITX3* is a determinant gene in eye development and is associated with congenital cataracts [14,15,16]. Although no CNV related to DSD was found, the results supported the etiology of congenital cataracts in this patient.
- **Patient #7**—A 1-year-old boy born with atypical genitalia (normal penile length, perineal hypospadias, bifid and hypodeveloped scrotum, and bilateral cryptorchidism) and imperforate anus. The patient presented dysmorphic facial and body features (Table 1). A sensorineural hearing loss attributed to a neonatal meningitis episode was detected. At the age of 3, an hCG stimulation test was normal. He underwent surgical correction of imperforate anus and ductus arteriosus persistence in the first year of life; and video-laparoscopy right gonad orchiopexy later in life; the left gonad was not found. At the age of 17, on his last follow-up visit, he had full pubertal development (Tanner V), micropenis (length of 7.5 cm; Z score: −4.2), topical urethra, and non-palpable gonads. Previous androgen replacement therapy was denied. A 46,XY del10q karyotype was revealed. The SNP array confirmed an 11.6 Mb deletion at the 10q25.3-q26.2 region (Figure 1F) and a 10q26 deletion syndrome (OMIM #609625) was made. Among the genes contained in the deleted 10q region (Table 2), the EMX2 (10q26.11) and FGFR2 (10q26.12) genes have been associated with 46,XY gonadal dysgenesis phenotype and are responsible for the atypical genitalia observed in this patient. The other features of facial dysmorphism, NPMD, congenital heart defects, and hearing loss could all be explained by this contiguous gene syndrome deletion.
- **Patient #15**—A 1-year-old boy born with atypical genitalia (balanic hypospadias, bilateral cryptorchidism, and hypodeveloped scrotum), microcephaly (cephalic perimeter (CP)—31 cm; Z-score: −2.7), body and facial dysmorphic features, and NPMD with the absence of corpus callosum (Table 2). At the age of 14, he underwent bilateral orchiopexy. Gonadal biopsy confirmed dysgenetic testis. At the age of 18, the patient underwent bilateral orchiectomy with insertion of testicular prostheses. A 46,XY karyotype without abnormalities was identified. The analysis of the SNP array revealed two CNVs classified as pathogenic; a 6.6 Mb duplication at 14q11.2-q12 and deletion of 12.7 Mb at 21p11.2-q21.3 (Figure 1C,D). Both CNVs contributed to the syndromic phenotype through a contiguous gene deletion syndrome.
- **Patient #22**—An 18-year-old man born with atypical genitalia (proximal hypospadias and bilateral cryptorchidism), and anal stenosis was referred for outpatient follow-up. He was born SGA and with microcephaly. Facial dysmorphisms, NPMD, ectopic right kidney, and partial deficiency of factors VII and X of coagulation were identified on follow-up (Table 1). Due to the lack of spontaneous puberty at age 16, exogenous testosterone was initiated. At age 17, he underwent male genitoplasty and bilateral orchiopexy, and bilateral gonadal biopsy revealed interstitial testicular fibrosis and absence of spermatogenesis. Karyotype analysis showed 46,XY (r13) (p11.2q34) and the SNP array identified a 10.9 Mb deletion at chromosome 13q33.1q34 responsible for a microdeletion syndrome (OMIM #619148) (Figure 2A). The haploinsufficiency of the *EFNB2* gene was probably responsible for the genital atypia and anorectal malformation. Haploinsufficiency of coagulation factor VII and X genes presented in this deletion may explain the clinical profile of partial deficiency of respective coagulation factors.

## 4. Discussion

Despite the accessibility of molecular techniques, including high-precision genomic arrays and large-scale parallel sequencing techniques, the molecular diagnosis of DSDs remains a challenge in daily clinical practice. The definitive molecular diagnosis of patients with DSD using these new techniques is suggested in approximately 40–50% of the cases [9,17,18]. The detection of CNVs associated with DSD has aided in the etiological diagnosis of syndromic and sporadic patients [18,19,20]. 

Currently, a small number of syndromic DSD patients are evaluated using the array methodology due to its rarity. This study shows the results of SNP-array analysis of 22 syndromic DSD patients from a single Brazilian reference center and contributes to amplify the knowledge of the features of patients’ cohort origin from a mixed-race population, usually underrepresented in the literature. SNP-array analysis helped to explain totally or partially the phenotype of six patients or approximately 27% of the studied patients, similar to what was found by Ledig et al., who reported 25% syndromic patients with pathogenic CNV [21]. 

Syndromic 46,XX DSD with a gain of Yp fragment, containing the SRY, may explain the DSD phenotype observed in two of six 46,XX patients of this cohort. The 46,XX testicular DSD condition is rare and affects 1: 20,000–30,000 live births [22,23]. The SRY translocation to the paternal X chromosome or autosomes constitutes the most frequent event associated with the testicular development in the majority of 46,XX testicular DSD patients (>80%) [24,25,26]. Usually, patients with 46,XX testicular DSD SRY (+) have typical male genitalia at birth and are diagnosed when adolescents or young adults seek medical attention due to gynecomastia, hypogonadism, or infertility without syndromic features [27]. Patients with 46,XX testicular DSD SRY (−) usually present genital atypia which leads to earlier diagnosis [28]. Several molecular etiologies have been related to this condition, characterizing the excess expression of male genes (*SOX9, SOX3, SOX10*) or reduced expression of female genes (*WNT4, RSPO1, NR2F2*) during the process of determining the embryonic gonad [28]. A differential diagnosis of this condition is the 46,XX ovo-testicular DSD (OT DSD), a rare abnormality of gonadal development [29]. The molecular aspects of both disorders are very similar, and these conditions may be a spectrum of the same disorder [30,31]. The deletion of 1.7 Mb in the 3q29 region in patient #2 overlapped with the genomic coordinates of the 3q29 deletion syndrome (OMIM #609425), which is characterized by NPMD associated with facial dysmorphisms such as those of our patient [32,33].

The 9 Mb deletion in Xp22.33 region in patient #5 overlapped the genomic coordinates of Aicardi syndrome [34,35,36,37]. Aicardi syndrome (OMIM %304050) is a neurodevelopmental disorder that affects mainly XX individuals with a prevalence of 1:100,000 live births. The classical syndrome triad is corpus callosum agenesis or hypoplasia, chorioretinal changes, and infantile spasms [38,39,40]; all of them were presented by patient #5. The pattern of genetic inheritance related to Aicardi syndrome remains unclear, but an X-linked inheritance has been suggested in several studies, leading to the Xp22 region as the one most frequently associated with the syndrome [41]. However, based exclusively on the results of the array, it is not possible to explain the condition of hypogonadotropic hypogonadism. Sequencing of ANOS1 excluded pathogenic variants in the preserved X allele gene copy. 

In the analysis of the 12 Kb deletion located at 10q24.32 found in patient #6, we identified the PITX3, which is expressed in neuronal cells, pituitary, and eyes. Pathogenic variants in PITX3 and Pitx3 have been associated with ocular and retinal malformations, and in humans more frequently with the presence of cataracts and malformations of the anterior eye segment [15,42,43,44]. 

Congenital cataracts affect 40 children for every 100,000 live births in the developed world [16]. Bidinost et al. described a Lebanese family of patients with delayed neuropsychomotor development and congenital cataracts associated with a PITX3 mutation [14]. PITX3 is a gene with the possibility of suffering haploinsufficiency with a pLI score = 0.813. Considering the absence of CNVs in the PITX3 associated with healthy individuals and the presence of CNVs previously reported in this gene with ocular malformation phenotype, the variant identified in the 10q24.32 region was probably responsible for the patient’s #6 ophthalmological phenotype. No CNV could explain the DSD phenotype. A custom panel of genes associated with DSD revealed the variant c.C1220G; p.P407R in the GATA4 classified according to the ACMG criteria as VUS. 

The 10q26 deletion syndrome (OMIM # 609625) diagnosed in patient #7 is described as a contiguous gene deletion syndrome with heterogeneous phenotypes. Facial dysmorphisms, delayed neuropsychomotor and behavioral development, short stature, malformations of extremities, cardiac and genitourinary malformations are features frequently presented by patients [45,46,47,48]. Atypical genitalia ranging from Micropenis and cryptorchidism to complete gonadal dysgenesis XY can be present [49,50,51]. Two genes (FGFR2 and EMX2) deleted in this CNV are strongly related to gonadal and genital development. 

The role of Fgfr2/Fgf9 in mice’s testicular determination is well demonstrated. It induces the proliferation of celomic epithelial cells leading to differentiation and maintenance of Sertoli cells. Fgfr2/Fgf9 acts to stimulate and maintaining Sox9 transcriptional levels [52,53]. Loss of functional variants in Fgfr2 compromised normal testicular differentiation causing disorganization of gonadal architecture with associated gonadal dysgenesis [52,53]. In humans, FGFR2 pathogenic variants, although rare, have been described in association with DSD and gonadal dysgenesis [54,55,56].

Emx2 knockout mice present with agenesis of kidneys, ureters, gonads, and absence of genital development [57,58]. Piard et al. compared the deleted regions of the short arm of chromosome 10 identified in several patients with atypical genitalia and established the smallest chromosomal region associated with this condition, and suggested EMX2 as the gene responsible for the phenotype [51,59,60]. Our report reinforces the involvement of FGFR2 and EMX2 in testicular determination in humans and its role in the DSD phenotype. Sensorineural deafness of patient #7 was associated with haploinsufficiency of two genes, HMX3 (OMIM 613380) and HMX2, both associated with vestibule and inner ear morphogenesis in mice and humans [47,59,61,62].

Two large CNVs (14q11.2-q12 6.6 Mb duplication and 21p11.2-q21.3 12.7 Mb deletion) detected in patient #15 were considered pathogenic. Duplications of CHD8 and SUPT16H genes located in our CNV are implicated in autistic spectrum phenotypes and NPMD [63]. Interstitial 21q deletion has a very heterogeneous phenotype ranging from severe neurological impairment with mental retardation, CNS malformation, congenital heart disease, and genitourinary malformation to milder conditions [64,65,66,67,68]. Patient #15, as well as patients with large interstitial deletions in the 21q described in the literature, have severe NPMD, microcephaly, and corpus callosum agenesis. Genital atypia and/or cryptorchidism, as observed in patient #15, have been reported in patients with 21q deletion syndrome, but no candidate gene related to this phenotype has been proposed [69].

The diagnose of 13q deletion syndrome (OMIM #613884) was made for patient #22 with the 10.9 Mb deletion in the 13q33.1-q34 region [70,71,72,73,74]. Anorectal and genitourinary abnormalities and NPMD are common finding in 13q [74,75,76,77,78]. Haploinsufficiency of the EFNB2 gene is probably responsible for the DSD phenotype. In knockout mice for the Efnb2, a defect in cloacal septation, with severe hypospadias in male mice, was demonstrated [79]. In humans, the EFNB2 encodes an EFNB class ephrin that binds to EPHB4 and EPHA3 receptors and plays a crucial role in the processes of migration, repulsion, and adhesion that occur during neuronal, vascular, epithelial, and urinary system development [80,81,82,83,84]. The haploinsufficiency of the EFNB2 (pLI = 0.99) constitutes the main candidate condition causing anorectal and urogenital malformations in patient #22. Genes for coagulation factors VII and X were also deleted in this region and are responsible for the partial clinical deficiency of coagulation factors VII and X [85,86]. 

## 5. Conclusions

In conclusion, the SNP-array analysis allowed us to expand the detection rate of genomic imbalances not previously detected by the karyotype analysis in patients with syndromic DSDs. In this cohort, rare pathogenic CNVs were identified in 27% of the patients, establishing the molecular cause of two 46,XX DSD patients. In three 46,XY DSD patients, CNVs containing DSD candidate genes or regions were classified as pathogenic, which may justify the patients’ DSD phenotypes. Finally, one of the patients presented a probably pathogenic CNV, which was not associated with DSD condition but contributed to partially elucidate the patient’s syndromic phenotype. These results reinforce the utility of genome-wide copy number analysis in the clinical practice to clarify the diagnoses of syndromic DSD patients and reinforce the role of candidate DSD genes and chromosomal regions.

## Figures and Tables

**Table 1:** DSD classification, phenotypical abnormalities, and description of external and internal genitalia of syndromic DSD patients studied using SNP array.

Patient	DSD Classification	Social Sex	Phenotypic Abnormalities	External Genitalia at Birth	* EMS/** Prader	Mullerian Ducts Derivatives
1	46,XX DSD	F	Facial dysmorphism, NPMD, CHD, CLD	Clitoromegaly, partial labial fusion, abdominal gonads	0/II	+
2	46,XX testicular DSD	M	Facial dysmorphism, NPMD, CHD, CLD	Typical male genitalia	12	-
3	46,XX ovo-testicular DSD	F	Facial dysmorphism, NPMD, CLD	Clitoromegaly, partial labial fusion, single urogenital orifice, palpable inguinal gonad (L)	2.5/III	+
4	46,XX DSD	M	Facial dysmorphism, NPMD, CHD, CAKUT, CLD	Micropenis, proximal hypospadias, bifid scrotum, palpable inguinal gonad (R)	2.5	-
5	46,XX DSD	F	Facial dysmorphism, NPMD, ACNS, epilepsy, hypoacusia, microphthalmia, blindness, hypogonadotrophic hypogonadism, obesity, CLD	Clitoromegaly, partial labial fusion, single urogenital orifice, palpable inguinal gonad (R)	2.5/III	-
6	46,XY DSD	M	Facial dysmorphism, congenital cataracts, NPMD, CLD	Micropenis, distal hypospadia, non-palpable gonads	5.5	-
7	46,XY DSD del 10q	M	Facial dysmorphism, NPMD, hypoacusia, SGA, CHD, ARM, CLD including incomplete cleft palate, ogival palate, mammary hypertelorism, single palmar fold and flat feet with the second curved toe	Micropenis, proximal hypospadia, bifid scrotum, non-palpable gonads	5.0	-
8	46,XY DSD	M	Facial dysmorphism, NPMD, CLD	Micropenis, glandular hypospadia, topic gonads	8.0	-
9	46,XY DSD	F	Facial dysmorphism, NPMD, epilepsy, ARM, obesity, CLD	Phallus agenesis, topic gonads	9.0	-
10	46,XY DSD	M	Facial dysmorphism, NPMD, premature, CAKUT, abdominal muscle malformation, ARM, CLD	Hemiphallus, non-palpable gonads	1.0	-
11	46,XY DSD	M	Facial dysmorphism, NPMD, SGA, premature birth, microcephaly, CLD	Micropenis, proximal hypospadias, bifid scrotum, bilateral palpable inguinal gonads	3.0	-
12	46,XY DSD	M	Facial dysmorphism, NPMD, CHD, SGA, CLD, joint hypermobility	Proximal hypospadia, bifid scrotum, bilateral palpable inguinal gonads	9.0	-
13	46,XY DSD del 1q	M	Facial dysmorphism, NPMD, CLD, microcephaly, premature birth	Midshaft hypospadias, non-palpable gonads	8.0	-
14	46,XY DSD t(3;9)	M	Facial dysmorphism, NPMD, SGA, CHD, CLD	Midshaft hypospadias, non-palpable gonads	9.0	-
15	46,XY DSD	M	Facial dysmorphism, NPMD, ACNS, microcephaly, CLD	Balanic hypospadias, palpable inguinal gonad (L)	9.5	-
16	47,XYY DSD	F	Facial dysmorphism, NPMD, thoracic malformation, CLD	Normal clitoris, partial labial fusion, non-palpable gonads	2.5	-
17	46,XY DSD	M	Facial dysmorphism, craniosynostosis, NPMD, premature birth, primary adrenal insufficiency	Curved penis, non-palpable gonads	11	-
18	46,XY DSD	M	Facial dysmorphism, NPMD, premature birth, CHD	Penoscrotal transposition, proximal hypospadias, topic gonads	10	-
19	46,XY DSD	M	Facial dysmorphism, NPMD	Micropenis, penoscrotal transposition, proximal hypospadias, topic gonad (R), palpable inguinal gonad (L)	6.5	-
20	46,XY DSD	M	Facial dysmorphism, NPMD, ARM, CLD	Proximal hypospadias, topic gonads	10	-
21	46,XY DSD	M	Facial dysmorphism, NPMD, ACNS, obesity	Non-palpable gonads	10.5	-
22	46,XY DSD	M	Facial dysmorphism, NPMD, microcephaly, SGA, ARM, MR	Proximal hypospadias, non-palpable gonads	8.5	-

EMS: external genitalia masculinization score, F: female, M: male, R: right, L: left, NPMD: neuropsychomotor developmental delay, SGA: small for gestational age, ARM: anorectal malformation, CHD: congenital heart defects, CLD: congenital limb defects, ACNS: anatomical central nervous system defects, CAKUT: congenital anomalies of the kidney and the urinary tract. * EMS score for 46,XY DSD patients or patients with male social sex. ** Prader scale for 46,XX patients with female social sex.

**Table 2:** Genomic location of pathogenic/probably pathogenic CNVs and gene content with OMIM reference number.

Patient	CNV	Affected Genes (OMIM Number)	Classification
**2**	**Yp11.31** (2,661,306–9,690,184) × 1	*SRY (480000), RPS4Y1 (470000), ZFY (490000), TGIF2LY (400025), PCDH11Y (400022), AMELY (410000), TBL1Y (400033), PRKY (400008), TSPY1 (480100)*	Pathogenic
**2**	**3q29** (195,677,895–197,413,261) × 1	*TFRC (190010), SLC51A (612084), PCYT1A (123695), RNF168 (612688), WDR53 (615110), NRROS (615322), PIGX (610276), PAK2 (605022), SENP5 (612845), NCBP2 (605133), PIGZ (611671), MFI2 (155750), DLG1 (611014), BDH1 (603063), KIAA0226 (613516)*	Pathogenic
**5**	**Yp11.31** (2,650,424–9,768,860) × 1	*SRY (480000), RPS4Y1 (470000), ZFY (490000), TGIF2LY (400025), PCDH11Y (400022), AMELY (410000), TBL1Y (400033), PRKY (400008), TSPY1 (480100)*	Pathogenic
**5**	**Xp22.33** (2,693,466–11,724,896) × 1	*XG (300879), GYG2 (300198), ARSD (300002), ARSE (300180), ARSH (300586), ARSF (300003), MXRA5 (300938), PRKX (300083), NLGN4X (300427), VCX3A (300533), PUDP (306480), STS (300747), VCX (300229), PNPLA4 (300102), VCX2 (300532), ANOS1 (300836), FAM9A (300477), FAM9B (300478), TBL1X (300196), GPR143 (300808), SHROOM2 (300103), CLCN4 (302910), MID1 (300552), HCCS (300056), ARHGAP6 (300118), AMELX (300391)*	Pathogenic
**6**	**10q24.32** (103,988,265–104,000,307) × 1	*PITX3 (602669), ELOVL3 (611815)*	Likely pathogenic
**7**	**10q25.3-q26.2** (118,087,298–129,753,712) × 1	*PNLIP (246600), PNLIPRP1 (604422) PNLIPRP2 (604423), HSPA12A (610701), KIAA1598 (611171), VAX1 (604294), KCNK18 (613655), SLC18A2 (193001), PDZD8 (614235), EMX2OS (607637), EMX2 (600035), RAB11FIP2 (608599), CASC2 (608598), PRLHR (600895), NANOS1 (608226), EIF3A (602039), PRDX3 (604769), GRK5 (600870), RGS10 (602856), TIAL1 (603413), BAG3 (603883), INPP5F (609389), MCMBP (610909), WDR11 (606417), FGFR2 (176943), ATE1 (607103), NSMCE4A (612987), TACC2 (605302), PLEKHA1 (607772), ARMS2 (611313), HTRA1 (602194), DMBT1 (601969), PSTK (611310), IKZF5 (606238), ACADSB (600301), HMX3 (613380), HMX2 (600647), BUB3 (603719), GPR26 (604847), CHST15 (608277), OAT (613349), FAM175B (611144), ZRANB1 (611749), CTBP2 (602619), MMP21 (608416), UROS (606938), BCCIP (611883), DHX32 (607960), FANK1 (611640), ADAM12 (602614), DOCK1 (601403), NPS (609513), PTPRE (600926)*	Pathogenic
**15**	**14q11.2-q12** (19,280,733–25,869,811) × 3	*OSGEP (610107), APEX (107748), PNP (164050), RNAE9 (614014), TRNAP1 (189930), TRL-AAG2-1 (189932), TRNAP2 (189931), TRNAT2 (189933), ANG (105850), RNASE4 (601030), EDDM3A (611580 ), FAM12B (611582), RNASE6 601981), RNASE1 (180440), RNASE3 (131398), RNASE2 (131410), METTL17 (616091), SLC39A2 (612166), NDRG2 (605272), TPPP2 (616956), RNASE7(612484), RNASE8(612485), SOLO (610018), ZNF219 (605036), HNRNPC (164020), RPGRIP1 (605446), SUPT16H (605012), CHD8 (610528), RAB2B (607466), TOX4 (614032), METTL3 (612472) SALL2 (602219), TRDC (186810), TRAC (186880), DAD1(600243), OXA1L (601066), SLC7A7 (603593), MRPL52 (611856), MMP14 (600754), LRP10 (609921), REM2 (616955), PRMT5 (604045), HAUS4 (613431), AJUBA 609066), PSMB5 (600306), PSMB11 (611137), CDH24 (618599), ACIN1(604562), CEBPE (600749), SLC7A8 (604235), HOMEZ, KIAA1443 (608119), BCL2L2 (601931), PABPN1 (602279), SLC22A17 (611461), EFS (609906), IL25 (605658), CMTM5 (607888), MYH6 (160710),MIR208A (611116), MYH7 (160760), MHRT (616096),MIR208B (613613), NGDN, NGD (610777), THTPA (611612), ZFHX2 (617828), AP1G2 (603534), DHRS2 (615194), DHRS4AS1 (616925), DHRS4 (611596), DHRS4L2 (615196), DHRS4L1 (615195),* *LRRC16B (614716), CPNE6 (605688), NRL (162080), PCK2 (614095), ARVD3 (602086),* *DFNB5(600792), SPG32 (611252), DCAF11 (613317), FITM1 (612028), PSME1 (600654), PSME2 (602161), RNF31 (612487), IRF9 (147574), REC8L1 (608193), TM9SF1 (618965),TSSK4 (610711), CHMP4A (610051), NEDD8 (603171),GMPR2 (610781), TINF2 (604319), TGM1 (190195), RABGGTA (601905), DHRS1(610410), NOP9 (618308), CIDEB (604441), LTB4R2 (605773), LTB4R (601531), ADCY4 (600292), RIPK3 (605817), NFATC4 (602699), CBLN3 (612978), SDR39U1 (616162), CMA1 (118938), CTSG (116830), GZMH (116831), GZMB (123910), STXBP6 (607958)*	Pathogenic
**15**	**21p11.2-q21.3** (14,613,203–27,328,175) × 1	*POTED (607549), LIPI (609252), RBM11 (617937), ABCC13 (608835), STCH (601100), SAMSN1 (607978), NRIP1 (602490), USH1E (602097), MIR99AHG (615964), MIR99A (614509), MIRLET7C (612144), MIR125B2 (610105), CXADR (602621), BTG3 (605674), CHODL (607247), TMPRSS15 (606635), NCAM2 (602040), MIR155(609337), MRPL39 (611845), JAM2 (606870), ATP5PF (603152), GABPA (600609), APP (104760)*	Pathogenic
**22**	**13q33.1-q34** (104,205,799–115,107,733) × 1	*DAOA-AS1 (607415), DAOA (607408), EFNB2 (600527), ARGLU1 (614046), LIG4 (601837), TNFSF13B (603969), MYO16 (615479), IRS2 (600797), COL4A1 (120130), COL4A2 (120090), NAXD (615910), CARS2 (612800), ING1 (601566), ARHGEF7 (605477), SOX1 (602148), ATP11A (605868), MCF2L (609499), F7 (613878), F10 (613872), PROZ (176895), PCID2 (613713), CUL4A (603137), LAMP1 (153330), ADPRHL1 (610620), TFDP1 (189902), ATP4B (137217), GRK1 (180381), GAS6 (600441), RASA3 (605182), CDC16 (603461), UPF3A (605530), CHAMP1 (616327)*	Pathogenic

Reference Consortium Human Build 37 (GRCh37)/hg19.

## Data Availability

The data that support the findings of the study are not publicly available due to individual privacy issues but are available from the corresponding author on reasonable request.
